# Illumination of a Vision 2020—Urinary Based Biomarkers for Bladder Cancer on the Way to Clinical Decisions—Dream or Nightmare?

**DOI:** 10.3390/ijms21051694

**Published:** 2020-03-02

**Authors:** Thorsten H. Ecke, Thomas Otto

**Affiliations:** 1 Department of Urology, HELIOS Hospital Bad Saarow, D-15526 Bad Saarow, Germany; 2 Department of Urology, Lukaskrankenhaus Neuss, D-41464 Neuss, Germany; 3 University Hospital Essen, D-45147 Essen, Germany

Bladder cancer is one of the most frequent malignancies worldwide [1]. For decades, it has been known that bladder cancer is heterogenous and therefore new markers are still needed for many different questions concerning diagnosis and therapy. Tumor grade and stage alone are not accurate in predicting the biological behavior and thus guiding the choice of treatment, especially in high risk cases [2,3,4].

Keeping the immense costs for bladder cancer patients from diagnosis to death with a calculation between US$96,000 and $187,000 in 2001 in mind, cheaper tests than cytology and tests preventing expensive and painful cystoscopies could help in reducing costs in the healthcare system [5,6]. However, additional information on risk profiles as well as personalized therapies are needed to reduce costs.

Up to date, no bladder cancer markers have been recommended in international guidelines, though cytology is still recommended in diagnostics [7]. Nevertheless, there are three interesting urinary-based fast tests that detect proteins on the market: UBC^®^, *rapid* test, BTA stat, and NMP22 [8,9,10]. All of these markers can show more or less high sensitivities in total or in some subgroups of bladder cancer, but none of them can be included into a routine or recommended in international guidelines. The lack of such meta-analysis lies in the problem that such studies are difficult to compare as test systems change during the years and studies differ significantly between each other regarding aim, material, and comparison. Therefore, the need of a study comparing these fast tests in comparison to the old gold standard cytology following the Paris system is needed [11]. On the other hand, in multicenter studies with a high volume of samples, the UBC^®^ *rapid* test is close to the sensitivity of cytology, especially for high grade tumors [12]. It seems that urinary based fast tests that detect proteins could be the way to replace cytology, which is still a subjective method [13].

Referring again to the European Association of Urology (EAU) guidelines, we can observe that positive results of cytology as well as UroVysion (FISH), NMP22, FGFR3/TERT, and microsatellite analysis in patients with negative cystoscopy and upper tract work-up may identify patients who more likely to experience disease and possible progression [7]. Most molecular tests are very expensive and difficult to analyze, but even in that field of diagnostics, there have been attempts to produce commercially available tests with high sensitivity and specificity at lower costs than in previous years. Mutations of TERT and FGFR3 can easily be detected by Uromonitor, a urinary based fast test that showed high sensitivities and specificities in an actual study [14].

Clinical evidence and molecular studies suggest that there are two pathways in human bladder carcinogenesis: the pTa pathway and the CIS (carcinoma in situ) pathway [15]. In most cases, pTa tumors are low-grade, even high-grade in a few cases, and they often recur, but rarely progress to lamina propria-invasive (pT1) and muscle-invasive tumors (pT2-T4), whereas CIS are high-grade by definition and are thought to be the most common precursor of invasive tumors. A urinary-based assay that can diagnose bladder cancer confined by the urothelium/CIS could fulfil the criterion to differ between both. This model has also been confirmed by other publications in the past [16,17,18].

There is a clinical need for markers to determinate the recurrence and progression of bladder cancer; these markers will contribute to establishing better treatments for the individual patient. Molecular staging of urological tumors will allow for the selection of cases that will require systemic and/or target treatment [19,20].

Clinical needs in uro-oncology are related to diagnosis, prognosis, and treatment. Uro-oncology is diverse, since genitourinary tumors differ histologically in their origin and various clinical behavior [21].

In the past, more and more genetic and epigenetic markers could show its predictive and/or prognostic value regarding overall survival and even cancer specific survival. As an example of how a tumor marker can predict therapeutic success, the results of the phase 2 KEYNOTE-052 (NCT02335424) study showed how a marker could influence therapeutic decisions on one hand, and that negative marker results were also not a certain predictor of therapeutic success [22]. PD-L1 positive is defined as a combined positive score (CPS) ≥10. In patients with CPS <10 (*n* = 251), the overall response rate (ORR) was 20%, while in patients with CPS ≥10 (*n* = 110), the ORR was 47%. This shows that even patients with negative CPS could have the benefit of pembrolizumab in this case.

This Special Issue has been introduced with the aim of offering the possibility of publishing new research results in the field of bladder cancer basic research. While editing this Special Issue, we have learned that enormous enthusiasm is necessary move forward in bladder cancer research. In our eyes, bladder cancer is, on one hand, a very heterogenous malignancy, which is why it so difficult to focus on a single bladder cancer marker in diagnostic and follow-up. Due to the mass of all these markers, it is impossible to report them all. This Special Issue tries to highlight the role of bladder cancer markers in diagnosis and the most important biomarkers studied and recently reported. Due to the determination of recurrence and progression, markers will contribute to establishing better treatments for the individual patient. Molecular staging of urological tumors will allow for cases to be selected that will require systemic treatment. However, as above-mentioned at the moment, it is not clear if clinical decisions based on tumor markers are still a dream, or perhaps a real nightmare. However, is still necessary and more important than before to integrate the same objectives under basic and clinical research.

The editors thank all submitting authors for their efforts and time spent for each manuscript. The lead editor would like to thank all editors for the time spent in reviewing, assigning reviews, and commenting on the submitted manuscripts. As an editorial team, we hope that this Special Issue will prove useful in research work regarding bladder cancer in the future. Hopefully, many researchers will use any kind of art to improve their professional success to ameliorate diagnostics and therapy in bladder cancer!

## References

[1] Ferlay J., Steliarova-Foucher E., Lortet-Tieulent J., Rosso S., Coebergh J.W., Comber H., Forman D., Bray F. (2013). Cancer incidence and mortality patterns in Europe: Estimates for 40 countries in 2012. Eur. J. Cancer.

[2] Theodorescu D., Wittke S., Ross M.M., Walden M., Conaway M., Just I., Mischak H., Frierson H.F. (2006). Discovery and validation of new protein biomarkers for urothelial cancer: A prospective analysis. Lancet Oncol..

[3] Sanchez-Carbayo M., Cordon-Cardo C. (2007). Molecular alterations associated with bladder cancer progression. Semin. Oncol..

[4] Mhawech-Fauceglia P., Cheney R.T., Schwaller J. (2006). Genetic alterations in urothelial bladder carcinoma: An updated review. Cancer.

[5] Botteman M.F., Pashos C.L., Redaelli A., Laskin B., Hauser R. (2003). The health economics of bladder cancer: A comprehensive review of the published literature. Pharmacoeconomics.

[6] Mitra N., Indurkhya A. (2005). A propensity score approach to estimating the cost-effectiveness of medical therapies from observational data. Health Econ..

[7] Babjuk M., Bohle A., Burger M., Capoun O., Cohen D., Compérat E.M., Hernández V., Kaasinen E., Palou J., Rouprêt M. (2017). EAU Guidelines on Non-Muscle-invasive Urothelial Carcinoma of the Bladder: Update 2016. Eur. Urol..

[8] Lu P., Cui J., Chen K., Lu Q., Zhang J., Tao J., Han Z., Zhang W., Song R., Gu M. (2018). Diagnostic accuracy of the UBC((R)) Rapid Test for bladder cancer: A meta-analysis. Oncol. Lett..

[9] Guo A., Wang X., Gao L., Shi J., Sun C., Wan Z. (2014). Bladder tumour antigen (BTA stat) test compared to the urine cytology in the diagnosis of bladder cancer: A meta-analysis. Can. Urol. Assoc. J..

[10] Wang Z., Que H., Suo C., Han Z., Tao J., Huang Z., Ju X., Tan R., Gu M. (2017). Evaluation of the NMP22 BladderChek test for detecting bladder cancer: A systematic review and meta-analysis. Oncotarget.

[11] Barkan G.A., Wojcik E.M., Nayar R., Savic-Prince S., Quek M.L., Kurtycz D.F.I., Rosenthal D.L. (2016). The Paris System for Reporting Urinary Cytology: The quest to develop a standardized terminology. J. Am. Soc. Cytopathol..

[12] Ecke T.H., Weiss S., Stephan C., Hallmann S., Arndt C., Barski D., Otto T., Gerullis H. (2018). UBC((R)) Rapid Test-A Urinary Point-of-Care (POC) Assay for Diagnosis of Bladder Cancer with a focus on Non-Muscle Invasive High-Grade Tumors: Results of a Multicenter-Study. Int. J. Mol. Sci..

[13] Schmitz-Drager B.J., Todenhofer T., van Rhijn B., Pesch B., Hudson M.A., Chandra A., Ingersoll M.A., Kassouf W., Palou J., Taylor J. (2014). Considerations on the use of urine markers in the management of patients with low-/intermediate-risk non-muscle invasive bladder cancer. Urol. Oncol..

[14] Batista R., Vinagre J., Prazeres H., Sampaio C., Peralta P., Conceição P., Sismeiro A., Leão R., Gomes A., Furriel F. (2019). Validation of a Novel, Sensitive, and Specific Urine-Based Test for Recurrence Surveillance of Patients With Non-Muscle-Invasive Bladder Cancer in a Comprehensive Multicenter Study. Front. Genet..

[15] Spruck C.H., Ohneseit P.F., Gonzalez-Zulueta M., Esrig D., Miyao N., Tsai Y.C., Lerner S.P., Schmütte C., Yang A.S., Cote R. (1994). Two molecular pathways to transitional cell carcinoma of the bladder. Cancer Res..

[16] Van Rhijn B.W., van der Kwast T.H., Vis A.N., Kirkels W.J., Boevé E.R., Jöbsis A.C., Zwarthoff E.C. (2004). FGFR3 and P53 characterize alternative genetic pathways in the pathogenesis of urothelial cell carcinoma. Cancer Res..

[17] Bakkar A.A., Wallerand H., Radvanyi F., Lahaye J.B., Pissard S., Lecerf L., Kouyoumdjian J.C., Abbou C.C., Pairon J.C., Jaurand M.C. (2003). FGFR3 and TP53 gene mutations define two distinct pathways in urothelial cell carcinoma of the bladder. Cancer Res..

[18] Hernandez S., Lopez-Knowles E., Lloreta J., Kogevinas M., Jaramillo R., Amorós A., Tardón A., García-Closas R., Serra C., Carrato A. (2005). FGFR3 and Tp53 mutations in T1G3 transitional bladder carcinomas: Independent distribution and lack of association with prognosis. Clin. Cancer Res..

[19] Lopez-Beltran A., Montironi R. (2004). Non-invasive urothelial neoplasms: According to the most recent WHO classification. Eur. Urol..

[20] Montironi R., Lopez-Beltran A. (2005). The 2004 WHO classification of bladder tumors: A summary and commentary. Int. J. Surg. Pathol..

[21] Agarwal P.K., Black P.C., Kamat A.M. (2008). Considerations on the use of diagnostic markers in management of patients with bladder cancer. World J. Urol..

[22] Suzman D.L., Agrawal S., Ning Y.M., Maher V.E., Fernandes L.L., Karuri S., Tang S., Sridhara R., Schroeder J., Goldberg K.B. (2019). FDA Approval Summary: Atezolizumab or Pembrolizumab for the Treatment of Patients with Advanced Urothelial Carcinoma Ineligible for Cisplatin-Containing Chemotherapy. Oncologist.

